# Impact of comorbidities and extra-musculoskeletal manifestations on radiographic progression in ankylosing spondylitis

**DOI:** 10.1093/rheumatology/keag247

**Published:** 2026-05-09

**Authors:** Sizheng Steven Zhao, Nicholas R Harvey, Bora Nam, Zhixiu Li, Linda A Bradbury, Lianne S Gensler, B Paul Wordsworth, Michael M Ward, Michael H Weisman, Thomas J Learch, John D Reveille, Tae-Hwan Kim, Matthew A Brown

**Affiliations:** Centre for Musculoskeletal Research, University of Manchester, Manchester, UK; NIHR Manchester Biomedical Research Centre, Manchester University NHS Foundation Trust, Manchester, UK; Department of Medical and Molecular Genetics, King’s College London, London, UK; Department of Medical and Molecular Genetics, King’s College London, London, UK; Department of Rheumatology, Hanyang University Hospital for Rheumatic Diseases, Seoul, Korea; School of Public Health and Emergency Management, Southern University of Science and Technology, Shenzhen, China; Centre for Genomics and Personalised Health, Queensland University of Technology, Brisbane, Australia; Gold Coast University Hospital, Brisbane, Southport, Queensland, Australia; Department of Medicine, Division of Rheumatology, University of California San Francisco, San Francisco, CA, USA; NIHR Oxford Biomedical Research Centre, Oxford University Hospitals NHS Foundation Trust, Oxford, UK; National Institute of Arthritis and Musculoskeletal and Skin Diseases, National Institutes of Health, Bethesda, MD, USA; Division of Immunology and Rheumatology, Stanford University, USA; Department of Radiology, Cedars-Sinai Medical Center, Los Angeles, CA, USA; Division of Rheumatology, McGovern Medical School, The University of Texas Health Science Center, Houston, TX, USA; Department of Rheumatology, Hanyang University Hospital for Rheumatic Diseases, Seoul, Korea; Department of Medical and Molecular Genetics, King’s College London, London, UK; Genomics England, London, UK

**Keywords:** axial spondyloarthritis, comorbidity, extra-musculoskeletal manifestations, uveitis, psoriasis, inflammatory bowel disease, cervical spine, radiographic progression

## Abstract

**Objective:**

To examine whether baseline comorbidity burden and extra-musculoskeletal manifestations (EMMs)—psoriasis, uveitis, IBD—are associated with spinal radiographic progression in AS.

**Methods:**

We analysed participants fulfilling modified New York criteria with one or more lateral cervical or lumbar radiograph. Radiographic progression was quantified using the modified Stoke AS Severity Score (mSASSS), excluding score 1 at each vertebral corner (range 0–48). Comorbidity count (22 self-reported conditions: none, one, two, three or more) and ever-presence of each EMM at baseline were exposures. mSASSS change over time with exposure-time interactions was modelled using generalized estimating equations; coefficients were rescaled to represent mean difference in progression (units/10 years). Models adjusted for baseline mSASSS, sex, symptom duration, CRP, HLA-B27, smoking, TNF inhibitor use, and number of EMMs or comorbidity count. Secondary analyses examined potential sex- and segment-specific effects.

**Results:**

Among 1150 individuals (mean age 44 years; 75% male; 84% HLA-B27 positive), 3441 patient-years were analysed (median follow-up 2 years; median two radiographs). Compared with those with no comorbidities, progression was greater amongst patients with two (2.7 units/10 years; 95% CI 1.9–3.5) and three or more (2.3; 1.5–3.1) comorbidities. Uveitis (2.2 units/10 years; 1.3–3.0) and psoriasis (2.4 units/10 years; 1.4–3.5), but not IBD, were associated with greater progression. Sex-specific analyses suggested greater spinal progression in females than males with psoriasis. Cervical-predominant changes were seen with uveitis and psoriasis.

**Conclusion:**

Comorbidity burden, uveitis and psoriasis are independently associated with greater spinal radiographic progression in AS. These readily identifiable features may inform risk stratification and targeted management strategies.

Rheumatology key messages

- Higher comorbidity burden independently associates with spinal radiographic progression in AS.
- Uveitis and psoriasis associate with accelerated, predominantly cervical, structural progression.
- Spinal progression is greater in females with psoriasis than in males, a finding that warrants replication.

## Introduction

Axial SpA (axSpA) is an immune-mediated disease characterized by chronic inflammatory back, but can also involve extra-musculoskeletal manifestations (EMMs) such as anterior uveitis, IBD and psoriasis [1]. In the axial skeleton, pathological inflammatory lesions are, for many, followed by structural damage with ankylosis of the sacroiliac joints and spine [2]. AxSpA can be classified into radiographic axSpA, also known as AS (also known as radiographic axSpA), or non-radiographic axSpA, depending on the presence of definite radiographic sacroiliitis [3].

Radiographic progression is associated with reduced physical function and health-related quality of life [4]. Preventing structural progression is a key goal of treatment [5], and there is accumulating evidence that targeted therapies can reduce radiographic progression [6]. However, not all individuals progress. Progression from non-radiographic axSpA to AS has been estimated at 8.7% over 10 years in the DESIR cohort [2], while progression of ≥2 mSASSS units over 5 years was seen in 28% of a Swedish cohort [7].

Better understanding of the risk factors for radiographic progression may help identify high-risk individuals who could benefit from more intensive disease-modifying therapy, and facilitate enriched recruitment for clinical trials of radiographic progression. Certain factors—such as male sex, CRP and baseline damage—have been consistently associated with greater progression risk [8], but most studies to date have focused on identifying predictors among a broad set of clinical features, rather than focused investigations of specific features. For example, obesity has been associated with radiographic damage, but the role of general comorbidity burden is unknown. Among EMMs, uveitis has been inconsistently associated with radiographic progression [9, 10], and little is known about psoriasis and IBD. Among the studies identifying sex as a risk factor, few have reported sex-specific differences in detail [11]. We aimed to examine the association between (i) comorbidities and (ii) EMMs, any potential interactions with sex, on radiographic progression among a well-characterized international AS cohort.

## Methods

We used data from the Australo-Anglo-American Spondyloarthritis Consortium (TASC), a prospective, international study that captured detailed clinical, biomarker and imaging data on patients fulfilling the modified New York criteria for AS. Written informed consent was obtained from all cases, with approval from the relevant research ethics authorities at each participating centre. The overall programme was reviewed and approved by Metro South Hospital Research Ethics Committee (approval reference HREC/05/QPAH/221).

The analysis included participants with at least one lateral cervical or lumbar spine radiograph scored using the modified Stoke Ankylosing Spondylitis Spine Score (mSASSS). The baseline visit was defined as the earliest radiograph, which was obtained between 2002 and 2017; subsequent images contributed to follow-up, with data-lock (final radiograph) in May 2018.

### Radiographic scoring

All radiographs were centrally scored according to the mSASSS protocol by a single experienced reader unblinded for date. A modified version of mSASSS (hereafter simply referred to as mSASSS) was used, whereby a score of 0 was assigned to normal vertebral corners, erosion, sclerosis or squaring, 1 to a definite syndesmophyte and 2 to complete bridging [12]; that is, the original mSASSS scores of ≥1 were subtracted by 1. This modification aimed to reduce misclassification between scores of 0 and 1 of the original mSASSS, and is supported by the lack of operational definitions for erosion, squaring and sclerosis; the lack of face validity in combining osteo-proliferative and osteo-destructive changes; and the poor inter-rater reliability of the original 0 and 1 scores [12–15]. Where fewer than four vertebral corners were missing in either the cervical or lumbar segment, the segment mean was imputed for those corners; if four or more corners were absent, the corresponding vertebral segment score, and therefore the total score, was set to missing. The total (sum of the two segment scores) ranged from 0 to 48. The inter-reader correlation for total mSASSS using this approach is high (0.94–0.99) [12].

### Exposures

Baseline comorbidity burden was defined as a count of 22 self-reported conditions, namely hypertension, cardiovascular disease (comprising heart attack, angina, coronary bypass, stroke), heart failure, cardiac arrhythmia (including atrial fibrillation or pacemaker), valvular disease, airway disease (emphysema, bronchitis, asthma), sleep apnoea, cancer (including skin and haematological malignancies), any fracture, osteoporosis, stomach ulcer, gastro-oesophageal reflux disease, hepatobiliary disease, diverticulitis, depression, headache, peripheral neuropathy, kidney disease, diabetes, glaucoma, cataracts and obesity. No response was assumed to mean absence of that comorbidity. Comorbidity count was categorized as none, one, two, or three or more comorbidities.

Self-reported ever or never presence of baseline EMMs—acute anterior uveitis, psoriasis and IBD—were captured as a binary variable.

Given prior literature demonstrating associations between sex and radiographic progression, particularly differences across cervical and lumbar segments [11], we also estimated studied sex as a standalone ‘exposure’ to provide context for subsequent analyses that test potential interactions between sex and comorbidity/EMM.

### Covariates

Baseline covariates included baseline mSASSS, sex, symptom duration, CRP (mg/dl), HLA-B27 status, TNF inhibitor (TNFi) use and smoking status (never, former, current). Models of comorbidity were adjusted for the number of EMMs (0–3) and models of each EMM were adjusted for comorbidity count. Age was not included due to its high collinearity with symptom duration (i.e. baseline age minus age of symptom onset). HLA-B27 status was excluded because preliminary analyses showed no independent association with radiographic progression. In sensitivity analyses, ASDAS-CRP was used instead of CRP as a measure of inflammatory disease activity.

### Statistical analysis

The association between each exposure and radiographic progression over time was modelled using generalized estimating equations (GEE) with an exchangeable working correlation structure to account for within-patient clustering, and robust standard errors (to ensure valid inference under potential misspecification of the correlation structure).

Models used mSASSS as the dependent variable, and included time (years), the exposure and their interaction as independent variables, adjusted for covariates listed above. The estimate for the interaction term represents the difference in the rate (regression slope) of mSASSS change over time between exposure groups. To facilitate interpretation, coefficients for the interaction term were rescaled to express the mean change in mSASSS per 10 years. The availability of radiographs varied across individuals, and GEE provides unbiased estimates under the assumption that missing mSASSS data are missing at random—that is, the probability of missingness depends only on observed data, not on unobserved or missing values. Primary multivariable models used complete-case analysis, but all models were repeated using multiple imputation by chained equations for missing covariates (with estimates from 30 imputed sets combined with Rubin’s rules). To facilitate interpretation, predicted marginal means were estimated from each GEE model, holding covariates at their mean values, and used to plot the model-predicted trajectories of structural progression over 10 years across exposure groups.

In secondary analyses, we additionally tested interaction terms with sex in each model (i.e. a three-way interaction term between mSASSS, time and sex), and provide sex-stratified estimates to aid interpretation. To facilitate interpretation, three-way interaction for comorbidity used binary presence/absence of any comorbidity. Because prior research suggested sex-specific effects at cervical and lumbar segments [16], we repeated all models for cervical and lumbar mSASSS separately (adjusting for segment-specific baseline in models). Lastly, we performed explorative analyses to examine contributions of individual comorbidities.

We performed a series of sensitivity analyses to test model assumptions and validity. First, we replaced CRP with ASDAS as indicator of inflammatory disease activity. Second, models for each EMM were additionally adjusted for the baseline presence of the other two EMMs. Third, for analysis of each EMM, we added an indicator category for those with missing EMM data. Fourth, we repeated analyses without including obesity in comorbidity count; BMI has been associated with radiographic progression [17], is a key component of psoriatic disease pathology and may drive associations with comorbidity count. Fifth, we repeated all analyses after applying coarsened exact matching [18] on age and symptom duration; exact matching was not feasible as it removed the majority of individuals. Sixth, we additionally adjusted for baseline use of NSAIDs. Finally, where data were available, we aimed to replicate analyses using an independent Korean dataset with unmodified mSASSS; details of this dataset have previously been published reference [19]. Analyses were performed with Stata 17 (StataCorp, College Station, TX, USA).

## Results

Analyses included 1150 individuals with a mean age of 44 (SD 14) years, of whom 75% were male and 84% were HLA-B27 positive (Table 1). Follow-up was over 3441 patient-years, with a median follow-up of 2.1 years (3.9 years among those with repeat radiographs), and median of 2 (interquartile range 1, 3) radiographs per individual. Among 81% with available baseline data, 38% were receiving a TNFi.

**Table 1:** Baseline characteristics.

	Any comorbidity	No comorbidities	*P*-value	Uveitis	No uveitis	*P*-value	Psoriasis	No psoriasis	*P*-value	IBD	No IBD	*P*-value
*N*	829	321		218	450		78	588		46	622	
Age (years)	46.4 (14.2)	36.5 (11.7)	<0.001	44.7 (13.1)	41.0 (13.8)	0.001	46.3 (13.5)	41.9 (13.8)	0.008	49.0 (14.0)	41.9 (13.7)	<0.001
HLA-B27, *n* (%)	689 (84)	259 (83)	0.86	194 (90)	343 (78)	<0.001	53 (70)	482 (83)	0.004	35 (76)	501 (82)	0.32
Symptoms duration (years)	22.1 (14.0)	13.2 (10.2)	<0.001	21.4 (13.1)	16.4 (12.9)	<0.001	22.0 (14.2)	17.7 (13.1)	0.009	25.1 (14.0)	17.7 (13.1)	<0.001
Baseline mSASSS	12.2 (15.7)	6.8 (11.7)	<0.001	10.4 (15.1)	8.7 (13.3)	0.16	11.7 (15.1)	9.0 (13.8)	0.12	10.1 (13.9)	9.3 (14.1)	0.73
CRP (mg/dl)	1.0 (1.7)	1.0 (3.0)	0.64	0.9 (1.4)	1.1 (2.8)	0.34	0.7 (1.4)	1.0 (2.6)	0.24	0.8 (1.3)	1.0 (2.5)	0.67
ASDAS	5.6 (3.4)	4.8 (3.2)	0.002	4.7 (3.2)	5.4 (3.4)	0.045	5.1 (3.4)	5.2 (3.4)	0.85	6.0 (3.0)	5.2 (3.4)	0.18
BASDAI	4.1 (2.4)	3.6 (2.5)	0.010	3.7 (2.4)	4.0 (2.5)	0.16	3.8 (2.6)	3.9 (2.4)	0.87	4.2 (2.2)	3.8 (2.5)	0.42
Smoking status, *n* (%)												
Never smoked	231 (37)	97 (46)	<0.001	57 (37)	150 (45)	0.039	22 (42)	184 (43)	0.34	8 (24)	199 (44)	0.011
Previous smoker	300 (48)	67 (32)		76 (49)	123 (37)		25 (48)	175 (41)		22 (67)	181 (40)	
Current smoker	98 (16)	45 (22)		21 (14)	58 (18)		5 (10)	73 (17)		3 (9)	72 (16)	
TNFi use, *n* (%)	254 (39)	94 (34)	0.12	67 (36)	194 (46)	0.015	32 (47)	225 (42)	0.44	22 (54)	238 (42)	0.16

Data are presented as mean (s.d.) or *n* (%).

### Comorbidities

**Figure 1:**
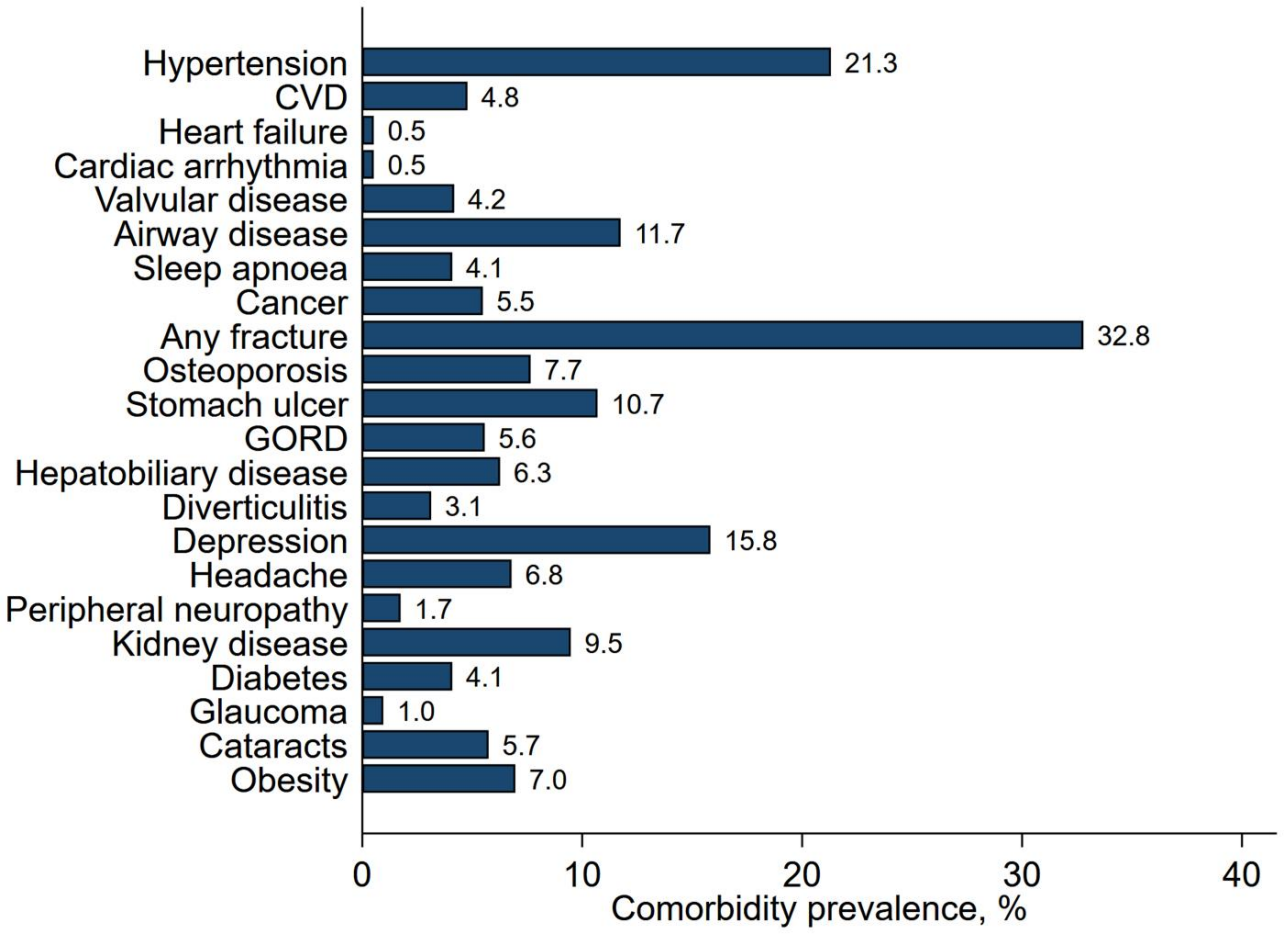
Prevalence of self-reported comorbidities. CVD: cardiovascular disease; GORD: gastro-oesophageal reflux disease.

At baseline, 310 (27%) had no comorbidities, 310 (27%) had one, 237 (21%) two and 282 (25%) three or more comorbidities; the median number of comorbidities was one (range 0–10). Fig. 1 shows the prevalence of each comorbidity. Compared with those without comorbidities, the group with any comorbidities were older, more often ever smokers, and had higher mean baseline mSASSS and ASDAS, but comparable CRP (Table 1). Sex-stratified comparisons are provided in Supplementary Table S1.

**Figure 2:**
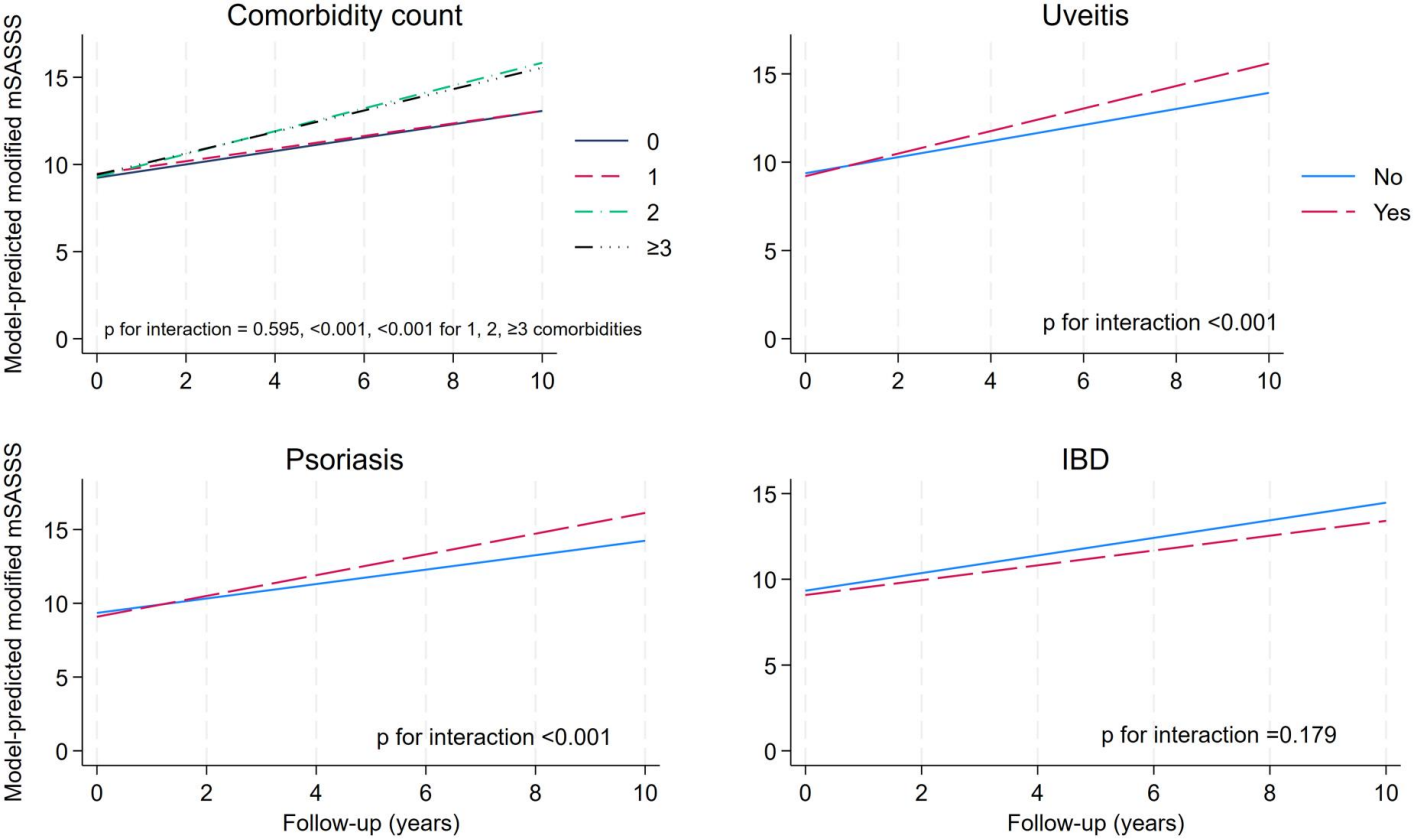
Radiographic progression over time across groups of comorbidity count and each extra-musculoskeletal manifestation. *P*-value of the interaction term pertains to the difference in slopes. mSASSS: modified Stoke Ankylosing Spondylitis Spine Score

**Figure 3:**
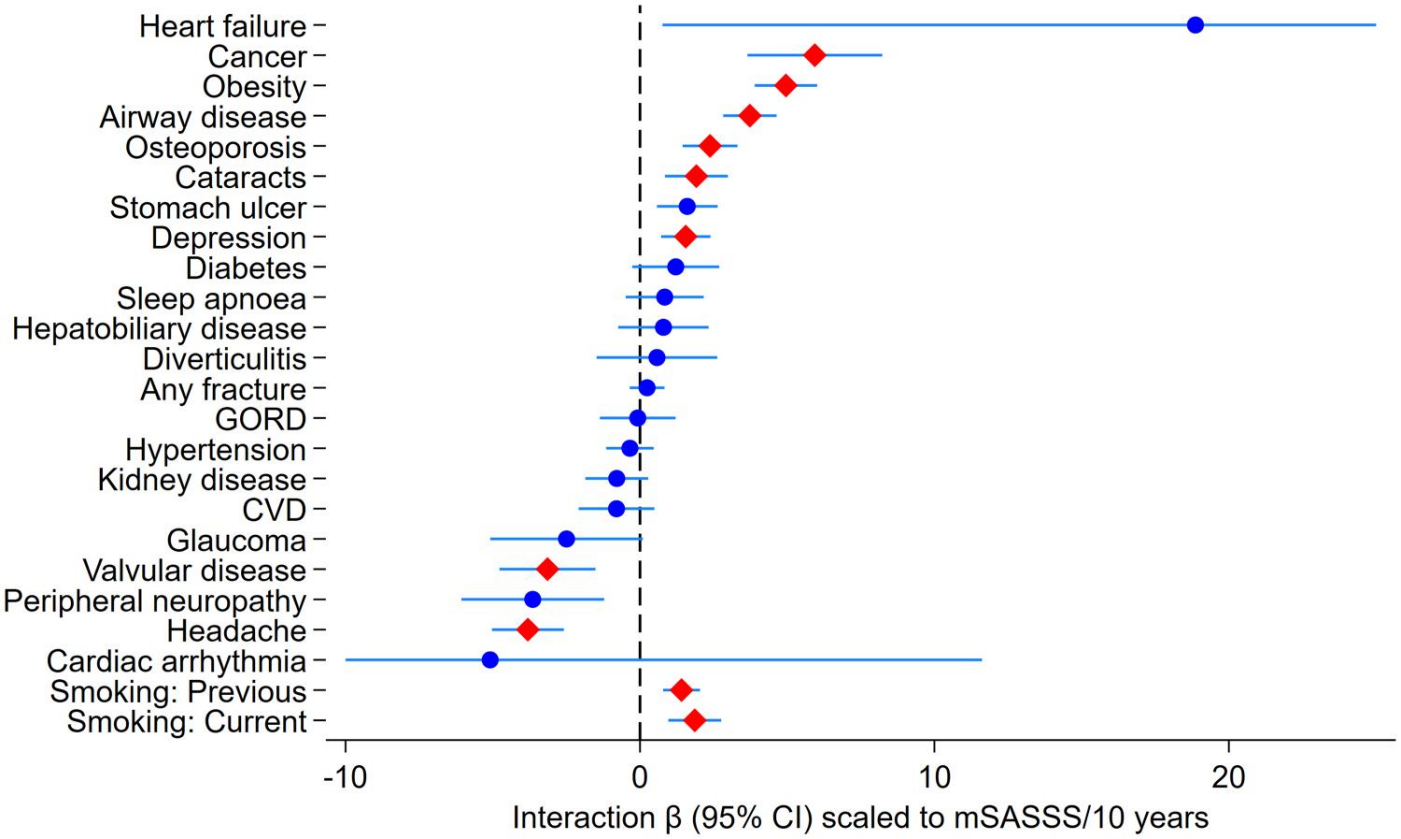
Explorative analysis of association between individual comorbidities and radiographic progression. β: regression coefficient; mSASSS: modified Stoke Ankylosing Spondylitis Spine Score; CVD: cardiovascular disease; GORD: gastro-oesophageal reflux disease. Red diamonds indicate statistically significant results after Bonferroni correction. Models adjusted for baseline mSASSS, sex, symptom duration, CRP, HLA-B27, TNF inhibitor use and smoking status

Radiographic mSASSS progression over time was greater with increasing comorbidity burden in adjusted models (Fig. 2). Compared with those without comorbidities, having one comorbidity was not associated with change in mSASSS (–0.2 mSASSS units over 10 years on average; 95% CI –1.0, 0.6; *P* = 0.60). In contrast, having either two (2.7 units/10 years; 95% CI 1.9, 3.5; *P* < 0.001) and three or more comorbidities (2.3 units/10 years; 95% CI 1.5, 3.1; *P* < 0.001) were associated with faster radiographic progression. Compared with those without comorbidities, having any comorbidities was associated with 1.8-unit higher mSASSS over 10 years (95% CI 1.1, 2.4; *P* < 0.001). In explorative analyses of individual comorbidities, cancer, obesity, airway disease, osteoporosis, cataracts and depression were associated with greater radiographic progression, while valvular disease and headache were associated with less radiographic progression (Fig. 3). Smoking (either past or current) was also associated with greater radiographic progression. Estimates were similar across sensitivity analyses (Supplementary Table S2).

### Extra-musculoskeletal manifestations

For EMMs, 668 participants completed questionnaires for uveitis (prevalence 32%), psoriasis (11%) and IBD (7%), respectively. Those with uveitis were older (45 *vs* 41 years; *P* = 0.001), more frequently HLA-B27 positive (90% *vs* 78%; *P* < 0.001) and more often ever-smokers (63% *vs* 55%; *P* = 0.039), but had similar baseline mSASSS (mean 9 *vs* 10 units; *P* = 0.16) and CRP (mean 1.0 *vs* 0.9 mg/dl; *P* = 0.34) (Table 2). The group with psoriasis were older (46 *vs* 42 years; *P* = 0.008) and less often HLA-B27 positive (70% *vs* 83%, 0.004), but otherwise comparable to those without psoriasis. Those with IBD were older (49 *vs* 42 years; *P* < 0.001) and more often ever-smokers (76% *vs* 56%; *P* = 0.011). Sex-stratified comparisons are provided in Supplementary Table S1.

**Table 2:** Difference in radiographic progression between groups with and without comorbidity and each extra-musculoskeletal manifestation.

	Comorbidity	Uveitis	Psoriasis	IBD
All	0.175 (0.113, 0.237)	0.217 (0.134, 0.300)	0.243 (0.137, 0.348)*	–0.064 (–0.207, 0.078)*
Male	0.228 (0.152, 0.303)	0.265 (0.164, 0.366)	0.183 (0.055, 0.312)	0.086 (–0.090, 0.263)
Female	0.219 (0.016, 0.421)	0.197 (0.011, 0.382)	0.449 (0.184, 0.714)	–0.279 (–0.584, 0.025)
Cervical spine	0.066 (0.011, 0.120)	0.105 (0.045, 0.165)	0.375 (0.286, 0.464)	–0.120 (–0.220, –0.019)
Lumbar spine	0.092 (0.054, 0.130)	0.037 (–0.020, 0.093)	–0.053 (–0.137, 0.031)	0.109 (0.007, 0.211)

* Estimate for GEE exposure–time interaction terms, that is, difference in radiographic progression between groups of each exposure. Significant (*P* < 0.1) three-way interaction with sex, suggesting difference in progression by sex.

Radiographic progression was statistically greater in the presence of uveitis (equivalent of 2.2 units difference in mSASSS over 10 years on average; 95% CI 1.3, 3.0; *P* < 0.001) and psoriasis (2.4 units/10 years; 95% CI 1.4, 3.5; *P* < 0.001), but not IBD (–0.6 units/10 years; 95% CI –2.1, 0.8; *P* = 0.38) (Fig. 2). Estimates were similar across sensitivity analyses (Supplementary Table S2).

To contextualize sex-specific effects, overall radiographic progression was greater in males than females (3.4 units/10 years; 95% CI 2.7, 4.1; *P* < 0.001). In secondary analyses testing sex as a potential effect modifier, we found significant interactions with sex in models of psoriasis (*P* = 0.01) and IBD (*P* = 0.01), but not comorbidity (*P* = 0.95) or uveitis (*P* = 0.67). In sex-stratified analyses to help interpret three-way interactions, progression appeared to be greater in females with psoriasis (4.5 units/10 years; 95% CI 1.8, 3.1; *P* = 0.001) than males (1.8 units/10 years; 95% CI 0.6, 3.1; *P* = 0.005). For IBD, sex-stratified estimates suggested greater progression in males, although wide CIs in both sexes limit interpretation (Table 2).

In separate models of cervical and lumbar spine, differences in mSASSS progression in uveitis and psoriasis appear to be driven by cervical spine scores, lumbar spine in IBD and no clear differences in the presence of comorbidities (Table 2). The apparent lack of progression in the lumbar spine may reflect a higher proportion of individuals with maximal baseline lumbar spine mSASSS among those with *vs* without uveitis (11% *vs* 5.5%; *P* < 0.001), although this was not observed for psoriasis (8.3% *vs* 7.0%; *P* = 0.5).

In the Korean replication dataset of 768 individuals (mean age 32 years, 90% male, 97% HLA-B27 positive, 6974 patient-years), only uveitis (prevalence 37%) was available for analysis. The presence of uveitis was associated with greater radiographic progression (4.5 units/10 years; 95% CI 3.0, 6.0; *P* < 0.001), adjusted for sex, symptom duration, smoking and HLA-B27 status. There was no interaction with sex, and segment-specific scores were not available for analysis.

## Discussion

Our results show that a higher comorbidity burden and the presence of either uveitis or psoriasis are each independently associated with faster spinal radiographic progression in AS. Individuals with these readily identifiable features therefore appear to be at greater risk of structural damage and may benefit from earlier or more intensive physical and pharmacological interventions. Segment-specific analyses suggest that the excess progression linked to uveitis and psoriasis is driven mainly by the cervical spine, whereas IBD is associated with greater change in the lumbar spine. We also observed faster overall spinal progression among females with psoriasis than males.

This is, to our knowledge, the first study to investigate the association between comorbidity burden and radiographic progression in AS. Previous studies have reported associations between BMI and syndesmophyte burden at baseline [17], and some with increased radiographic progression [7, 20], but neither in the context of other comorbidities or EMMs. Our findings suggest that individuals with greater comorbidity burden are at higher risk of structural progression, even after excluding obesity from comorbidity count and adjusting for other recognized risk factors. The mechanisms underlying this association remain unclear; there may be a latent factor common to adiposity and other comorbidities. Further research is warranted to identify which specific comorbid conditions may contribute most to this risk, and to elucidate potential causal pathways. This study was not powered to dissect the contributions of individual comorbidities.

The observed associations between individual comorbidities and radiographic progression should be regarded as exploratory and interpreted with caution. Some associations are plausibly explained by underlying disease severity; for example, more severe disease may increase both the risk of progression and the likelihood of detecting comorbidities such as osteoporosis or depression through more intensive follow-up. Others may be related to shared risk factors such as smoking, which could explain the links with airway disease and cancer. Apparent ‘protective’ associations are unlikely to be causal; for instance, there was markedly higher headache prevalence in females and sex may contribute to residual confounding. Similarly, valvular disease includes valve replacements, which may be more frequently performed in individuals with fewer risk factors for both surgery and progression. Finally, the current comorbidity data were self-reported and associations may also result from biased reporting and/or misclassification. Nonetheless, these findings generate hypotheses of potential interest that warrant confirmation in future, adequately powered studies—ideally incorporating sex-specific analyses.

The literature on associations between EMMs and radiographic progression has been inconsistent. A single-centre Korean study of 253 propensity score–matched ASAS-criteria patients showed lower odds [odds ratio (OR) 0.21; 95% CI 0.07, 0.67] of progression (mSASSS increase by ≥2 units) at 2 years in those with uveitis [9]. In contrast, analysis of the Observation Study of Korean SpA Registry (OSKAR), comprising 598 patients meeting the modified New York criteria, reported no difference in mSASSS at 5 years (5.49 with *vs* 6.29 without uveitis; *P* = 0.68) [10]. Discrepant results across each of these studies and ours may be due to differences in statistical methods and/or cohort characteristics. Both Korean studies assessed change between two timepoints rather than modelling progression longitudinally, which may not fully capture trajectory over time and may introduce selection bias. For example, the single-centre Korean study analysed only 61% of the total population with available radiographs. In a Swedish cohort of 204 patients, uveitis was numerically associated with progression (mSASSS increase ≥2) any time over a 5-year period (OR 1.97; 95% CI 0.90, 4.30) [7]. Analysis of the European Outcome in Ankylosing Spondylitis International Study (OASIS) cohort, comprising 216 individuals meeting modified New York criteria, reported no differences in mSASSS, over a mean of 8.3 years, for each EMM in GEE models [21]. The OASIS analysis did not specify use of an exposure–time interaction term [21]; if analyses were performed as reported, then the estimate for the uveitis variable in GEE reflects differences in mSASSS at the start of follow-up (which was also non-significant in our study), not mSASSS change over time.

We demonstrated the association between uveitis and increased radiographic progression in two independent cohorts, representing the largest sample sizes to date. Interestingly, a prior study showed that HLA-B27-positive uveitis is more severe when it co-occurs with axSpA compared with when it occurs in isolation [22]. These associations may be explained by a higher inflammatory liability, for example driven by greater burden of susceptibility alleles, which predisposes to more disease manifestations and greater severity within each manifestation. Uveitis may therefore serve as a marker of a more aggressive disease phenotype, and help identify individuals at higher risk of radiographic damage. Recognizing this subgroup may aid both in guiding treatment decisions and in enriching future clinical trials that use structural progression as an outcome.

Psoriasis is typically associated with more prominent peripheral joint involvement [23]. However, adequately powered studies of psoriasis and axial disease progression are lacking. Analysis of 88 early axSpA patients from the Italian SPACE cohort reported greater radiographic progression over 4 years among the 36% with psoriasis [24]. This was supported by higher mSASSS in those with psoriasis than without, at baseline (4 *vs* 1 mSASSS units) and at 4 years (7 *vs* 2 mSASSS units). However, these raw results contradict the lower odds (OR 0.18; 95% CI 0.04, 0.78) of progression (mSASSS change by ≥2 mSASSS units) in regression models. A conference abstract of 210 axSpA patients (13% with psoriasis) reported no statistically significant association with radiographic progression, though point estimates suggested a possible 2- to 3-fold increase in progression in the spine (OR 2.93; 95% CI 0.81, 10.6) and sacroiliac joints (OR 1.98; 95% CI 0.72, 5.43) [25]. Our findings support an independent association between psoriasis and radiographic progression, possibly greater in the cervical spine and among females, that warrant further replication.

The principal strength of this study is its large, international cohort and the application of statistical methods that optimize the use of longitudinal data. Nonetheless, several limitations should be acknowledged. First, radiographs were not systematically scored by more than one reader, which may introduce bias; however, as this was an ancillary analysis, the reader was unlikely to have been aware of participants’ exposure status in a way that would systematically influence scoring. We sought to address the primary source of inter-rater variability by applying the modified mSASSS, which has been shown in prior studies to reduce scoring variability [12]. As previously reported, pairwise inter-reader reliability in this study was high, with intraclass correlation coefficients ranging from 0.94 to 0.99 [12]. Because duplicate scoring was not available for this ancillary analysis, residual inter-rater variability cannot be excluded and is acknowledged as a limitation. Second, it was not possible to align any time-varying treatment data with dates of radiographs, which should be better addressed in future studies. Baseline TNFi use was comparable between participants with and without comorbidities or psoriasis, but lower among those with uveitis. Less intensive use of targeted therapies may have contributed to greater radiographic progression in the uveitis group, although this could be counterbalanced by their lower ASDAS scores—a known predictor of progression. Third, EMM ascertainment by self-report is susceptible to misclassification (e.g. non-specific rash as psoriasis or irritable bowel syndrome as IBD), which would inflate prevalence and noise, thus bias estimates towards the null. However, the prevalence of each EMM in our data closely resembles that reported in prior literature. Fourth, median follow-up was 3.9 years among those with repeat radiographs. Given that radiographic progression is slow, estimates—particularly modelled over longer time horizons—should be interpreted cautiously. Lastly, secondary analyses should be interpreted with caution given the number of statistical tests conducted, and warrant replication in independent cohorts.

In conclusion, comorbidity burden, uveitis and psoriasis are independently associated with accelerated spinal radiographic progression in AS. These readily identifiable clinical features may help stratify individuals at higher risk and inform the use of targeted physical and pharmacological interventions. Further research is needed to determine which comorbidities contribute most significantly to progression, to clarify underlying mechanisms, and to replicate the observed associations with psoriasis in independent cohorts.

## Supplementary Material

keag247_Supplementary_Data

## Data Availability

The individual-level data underlying this study are not publicly available due to ethical restrictions related to participant confidentiality and data protection. De-identified data may be made available to qualified researchers upon reasonable request for the purpose of scientific collaboration. Requests should be directed to the corresponding author and will be reviewed by the consortium partners in accordance with applicable institutional and regulatory requirements.
