# Supplementary material for: Impact of comorbidities and extra-musculoskeletal manifestations on radiographic progression in ankylosing spondylitis
Source: Rheumatology (Oxford). 2026 May 9;65(6):keag247. doi: 10.1093/rheumatology/keag247 (PMC13240999; doi:10.1093/rheumatology/keag247)
Supplement: keag247_Supplementary_Data [file keag247_supplementary_data.docx]

**Supplementary materials for:**

**Impact of comorbidities and extra-musculoskeletal manifestations on radiographic progression in ankylosing spondylitis**

Sizheng Steven Zhao^1,2,3^, Nicholas R Harvey^3^, Bora Nam^4^, Zhixiu Li^5,6^, Linda A Bradbury^7^, Lianne S Gensler^8^, B Paul Wordsworth^9^, Michael M Ward^10^, Michael H Weisman^11^, Thomas J Learch^12^, John D Reveille^13^, Tae-Hwan Kim^4^, Matthew A Brown^3,14^

1. Centre for Musculoskeletal Research, University of Manchester, Manchester, United Kingdom
2. NIHR Manchester Biomedical Research Centre, Manchester University NHS Foundation Trust, Manchester
3. Department of Medical and Molecular Genetics, King’s College London, London, United Kingdom
4. Hanyang University Hospital for Rheumatic Diseases, Seoul, Korea, Rep. of (South Korea)
5. School of Public Health and Emergency Management, Southern University of Science and Technology, Shenzhen, China
6. Queensland University of Technology, Centre for Genomics and Personalised Health, Brisbane, Australia
7. Translational Research Institute, University of Queensland Diamantina Institute, Brisbane, Australia
8. Department of Medicine, Division of Rheumatology, University of California San Francisco, San Francisco, United States of America
9. NIHR Oxford Biomedical Research Centre, Oxford University Hospitals NHS Foundation Trust , Oxford, United Kingdom.
10. National Institute of Arthritis and Musculoskeletal and Skin Diseases, National Institutes of Health, Bethesda, United States of America
11. Division of Immunology and Rheumatology, Stanford University, United States of America
12. Department of Radiology, Cedars-Sinai Medical Center, Los Angeles, California, United States of America
13. Division of Rheumatology, McGovern Medical School at The University of Texas Health Science Center, Houston, United States of America
14. Genomics England, London, United Kingdom

Correspondence: Professor Matthew A Brown. Department of Medical and Molecular Genetics, Faculty of Life Sciences and Medicine, King’s College London, London, SE1 9RT, United Kingdom; [matt.brown@kcl.ac.uk](mailto:matt.brown@kcl.ac.uk)

## **Supplementary Table S1. Baseline characteristics by sex across exposures.**

|  | **Male comorbidities** | | | **Female comorbidities** | | | **Male uveitis** | | | **Female uveitis** | | | **Male psoriasis** | | | **Female psoriasis** | | | **Male IBD** | | | **Female IBD** | | |
| --- | --- | --- | --- | --- | --- | --- | --- | --- | --- | --- | --- | --- | --- | --- | --- | --- | --- | --- | --- | --- | --- | --- | --- | --- |
|  | **Yes** | **No** | **p** | **Yes** | **No** | **p** | **Yes** | **No** | **p** | **Yes** | **No** | **p** | **Yes** | **No** | **p** | **Yes** | **No** | **p** | **Yes** | **No** | **p** | **Yes** | **No** | **p** |
| N | 616 | 241 |  | 213 | 80 |  | 151 | 358 |  | 67 | 92 |  | 60 | 448 |  | 18 | 140 |  | 35 | 475 |  | 11 | 147 |  |
| Age (years) | 47.1 (14.4) | 36.3 (12.0) | <0.001 | 44.3 (13.3) | 36.9 (11.0) | <0.001 | 46.0 (13.7) | 41.2 (14.1) | <0.001 | 41.7 (11.2) | 40.0 (12.6) | 0.40 | 47.5 (13.9) | 42.1 (14.1) | 0.006 | 42.2 (11.5) | 41.2 (12.5) | 0.74 | 51.4 (14.6) | 42.2 (14.0) | <0.001 | 41.3 (8.8) | 41.1 (12.7) | 0.96 |
| HLA-B27 | 518 (85%) | 198 (85%) | 0.98 | 171 (80%) | 61 (78%) | 0.70 | 136 (91%) | 278 (80%) | 0.002 | 58 (87%) | 65 (71%) | 0.018 | 42 (72%) | 369 (84%) | 0.028 | 11 (61%) | 113 (81%) | 0.057 | 26 (74%) | 389 (84%) | 0.15 | 9 (82%) | 112 (76%) | 0.67 |
| Symptoms duration | 22.8 (14.0) | 13.6 (10.4) | <0.001 | 19.9 (13.8) | 12.2 (9.8) | <0.001 | 22.8 (13.6) | 16.7 (12.8) | <0.001 | 18.3 (11.3) | 15.2 (13.5) | 0.14 | 23.7 (14.3) | 18.0 (13.2) | 0.003 | 16.2 (12.6) | 16.9 (13.0) | 0.84 | 27.0 (14.5) | 18.0 (13.1) | <0.001 | 19.3 (10.9) | 16.5 (13.0) | 0.49 |
| Baseline mSASSS | 14.7 (16.5) | 8.3 (12.8) | <0.001 | 5.0 (10.1) | 2.2 (5.9) | 0.020 | 13.0 (16.1) | 10.0 (14.1) | 0.042 | 4.8 (11.1) | 3.8 (8.1) | 0.54 | 13.2 (16.1) | 10.5 (14.5) | 0.20 | 6.5 (9.3) | 4.2 (9.7) | 0.35 | 12.5 (15.0) | 10.9 (14.9) | 0.56 | 2.3 (3.0) | 4.4 (9.6) | 0.50 |
| CRP (mg/dl) | 1.0 (1.6) | 0.9 (1.4) | 0.54 | 0.9 (1.9) | 1.4 (5.5) | 0.28 | 0.9 (1.5) | 0.9 (1.5) | 0.64 | 0.8 (1.3) | 1.5 (5.4) | 0.35 | 0.8 (1.6) | 0.9 (1.5) | 0.53 | 0.3 (0.2) | 1.3 (4.5) | 0.32 | 1.0 (1.4) | 0.9 (1.5) | 0.83 | 0.4 (0.4) | 1.3 (4.4) | 0.52 |
| ASDAS | 5.6 (3.4) | 4.6 (3.2) | <0.001 | 5.5 (3.5) | 5.3 (3.3) | 0.62 | 4.8 (3.2) | 5.3 (3.4) | 0.18 | 4.7 (3.3) | 5.9 (3.5) | 0.069 | 4.9 (3.2) | 5.1 (3.3) | 0.65 | 5.8 (3.9) | 5.5 (3.5) | 0.74 | 5.9 (3.1) | 5.1 (3.4) | 0.23 | 6.2 (2.9) | 5.4 (3.5) | 0.54 |
| BASDAI | 4.0 (2.4) | 3.4 (2.5) | 0.015 | 4.5 (2.5) | 4.2 (2.5) | 0.43 | 3.6 (2.3) | 3.8 (2.5) | 0.49 | 3.8 (2.5) | 4.8 (2.4) | 0.027 | 3.6 (2.5) | 3.7 (2.4) | 0.82 | 4.5 (3.1) | 4.6 (2.5) | 0.98 | 4.0 (2.1) | 3.7 (2.4) | 0.50 | 4.9 (2.6) | 4.4 (2.5) | 0.62 |
| Never smoked | 160 (34%) | 73 (44%) | <0.001 | 71 (45%) | 24 (56%) | 0.43 | 35 (33%) | 115 (44%) | 0.005 | 22 (47%) | 35 (49%) | 0.79 | 15 (38%) | 135 (41%) | 0.36 | 7 (54%) | 49 (46%) | 0.75 | 5 (20%) | 147 (43%) | 0.010 | 3 (38%) | 52 (48%) | 0.78 |
| Previous smoker | 235 (50%) | 53 (32%) |  | 65 (41%) | 14 (33%) |  | 59 (55%) | 95 (37%) |  | 17 (36%) | 28 (39%) |  | 20 (51%) | 134 (41%) |  | 5 (38%) | 41 (39%) |  | 18 (72%) | 140 (41%) |  | 4 (50%) | 41 (38%) |  |
| Current smoker | 75 (16%) | 40 (24%) |  | 23 (14%) | 5 (12%) |  | 13 (12%) | 49 (19%) |  | 8 (17%) | 9 (12%) |  | 4 (10%) | 57 (17%) |  | 1 (8%) | 16 (15%) |  | 2 (8%) | 56 (16%) |  | 1 (12%) | 16 (15%) |  |
| TNFi use | 180 (38%) | 74 (36%) | 0.60 | 74 (42%) | 20 (27%) | 0.032 | 46 (37%) | 148 (45%) | 0.11 | 21 (34%) | 46 (53%) | 0.026 | 21 (41%) | 171 (42%) | 0.89 | 11 (65%) | 54 (42%) | 0.075 | 16 (53%) | 181 (42%) | 0.24 | 6 (55%) | 57 (42%) | 0.43 |

## **Table S2. Sensitivity analysis results.**

|  | **Comorbidity (yes/no)** | **Comorbidity count***  **(0, 1, 2, ≥3)** | **Uveitis** | **Psoriasis** | **IBD** |
| --- | --- | --- | --- | --- | --- |
| **Primary analysis** | 0.175 (0.113, 0.237) | 0.131 (0.059, 0.202)  0.152 (0.075, 0.229)  0.246 (0.167, 0.326) | 0.217 (0.134, 0.300) | 0.243 (0.137, 0.348) | -0.064 (-0.207, 0.078) |
| **Sensitivity analysis 1:** Replace CRP with ASDAS | 0.195 (0.129, 0.261) | 0.021 (-0.063, 0.105) 0.174 (0.091, 0.258) 0.371 (0.287, 0.455) | 0.332 (0.227, 0.436) | 0.728 (0.618, 0.837) | -0.094 (-0.303, 0.115) |
| **Sensitivity analysis 2:** Additionally adjust EMM models for the other two baseline EMMs | na | na | 0.234 (0.157, 0.311) | 0.252 (0.140, 0.363) | -0.040 (-0.175, 0.095) |
| **Sensitivity analysis 3:** Include missing-indicator category for EMM status | na | na | 0.216 (0.146, 0.286) | 0.243 (0.145, 0.340) | -0.066 (-0.183, 0.051) |
| **Sensitivity analysis 4:** Exclude obesity from comorbidity count | 0.169 (0.107, 0.231) | 0.131 (0.059, 0.202) 0.152 (0.075, 0.229) 0.246 (0.167, 0.326) | 0.234 (0.158, 0.310) | 0.252 (0.143, 0.362) | -0.040 (-0.173, 0.092) |
| **Sensitivity analysis 5:** Coarsened exact matching on age and symptom duration | 0.163 (0.099, 0.227) | -0.004 (-0.031, 0.024) 0.263 (0.203, 0.323) 0.168 (0.134, 0.203) | 0.192 (0.103, 0.280) | 0.191 (0.063, 0.319) | -0.071 (-0.168, 0.027) |
| **Sensitivity analysis 6:** Additionally adjust for baseline NSAID use | 0.286 (0.199, 0.372) | 0.172 (0.064, 0.280) 0.402 (0.299, 0.505) 0.226 (0.121, 0.331) | 0.217 (0.102, 0.332) | 0.231 (0.088, 0.373) | 0.000 (-0.176, 0.176) |
| Estimate for GEE exposure-time interaction terms, that is, difference in radiographic progression between groups of each exposure  *results shown with 0 as referent. | | | | | |
